# Extracellular nucleotides mediate viral central nervous system infections: Key alarmins of neuroinflammation and neurodegeneration

**DOI:** 10.4103/NRR.NRR-D-24-01464

**Published:** 2025-05-06

**Authors:** Raíssa Leite-Aguiar, Elaine Paiva-Pereira, Robson Coutinho-Silva, Cláudia Pinto Figueiredo, Luiz Eduardo Baggio Savio

**Affiliations:** 1 Laboratório de Neuroimunologia, Instituto de Biofísica Carlos Chagas Filho, Universidade Federal do Rio de Janeiro, Rio de Janeiro, Brazil; 2 Laboratório de Imunofisiologia, Instituto de Biofísica Carlos Chagas Filho, Universidade Federal do Rio de Janeiro, Rio de Janeiro, Brazil; 3 Faculdade de Farmácia, Universidade Federal do Rio de Janeiro, Rio de Janeiro, Brazil

**Keywords:** adenosine triphosphate, dengue, Epstein-Barr virus, herpes simplex virus type 1, human immunodeficiency virus type 1, neurodegenerative diseases, neurotropic infections, purinergic signaling, severe acute respiratory syndrome coronavirus 2, virus, Zika

## Abstract

Recent increases in infectious diseases affecting the central nervous system have raised concerns about their role in neuroinflammation and neurodegeneration. Viral pathogens or their products can invade the central nervous system and cause damage, leading to meningitis, encephalitis, meningoencephalitis, myelitis, or post-infectious demyelinating diseases. Although neuroinflammation initially has a protective function, chronic inflammation can contribute to the development of neurodegenerative diseases. Mechanisms such as protein aggregation and cellular disturbances are implicated with specific viruses such as herpes simplex virus type 1 and Epstein-Barr virus being associated with Alzheimer’s disease and multiple sclerosis, respectively. Extracellular nucleotides, particularly adenosine triphosphate and its metabolites are released from activated, infected, and dying cells, acting as alarmins mediating neuroinflammation and neurodegeneration. When viruses infect central nervous system cells, adenosine triphosphate is released as an alarmin, triggering inflammatory responses. This process is mediated by purinergic receptors, divided into two families: P1, which responds to adenosine, and P2, activated by adenosine triphosphate and other nucleotides. This review highlights how specific viruses, such as human immunodeficiency virus type 1, Theiler’s murine encephalomyelitis virus, herpes simplex virus type 1, Epstein-Barr virus, dengue virus, Zika virus, and severe acute respiratory syndrome coronavirus 2, can initiate inflammatory responses through the release of extracellular nucleotides, particularly adenosine triphosphate, which act as critical mediators in the progression of neuroinflammation and neurodegenerative disorders. A better understanding of purinergic signaling pathways in these diseases may suggest new potential therapeutic strategies for targeting neuroinflammation to mitigate the long-term consequences of viral infections in the central nervous system.

## Introduction

In recent years, there has been a significant increase in infectious diseases affecting the central nervous system (CNS) worldwide (Stephenson et al., 2018). Various viruses from different families, including DNA and RNA viruses, can infect the CNS, leading to neuroinflammatory conditions such as meningitis, encephalitis, or meningoencephalitis (Klein et al., 2017). These viruses can be classified based on their interaction with the nervous system as either classically neurotropic, such as rabies virus, herpes simplex virus (HSV), poliovirus, and Japanese encephalitis virus, which frequently infect neural tissues and cause neurological diseases, or occasionally neuroinvasive, such as severe acute respiratory syndrome coronavirus 2 (SARS-CoV-2), human immunodeficiency virus (HIV), Chikungunya virus, and dengue virus, which primarily target other systems but may invade the CNS under certain conditions. Once within the CNS, the resulting infection often triggers neuroinflammation, where the activation of microglia and astrocytes aims to resolve the infection and maintain tissue homeostasis by producing and releasing cytokines and chemokines. However, when inflammation becomes persistent, it can lead to cellular dysfunctions that significantly contribute to neurodegeneration (Yang and Zhou, 2019).

This chronic inflammatory state is a hallmark of neurodegenerative disorders, where external factors and internal cellular disturbances can drive inflammation. Mechanisms such as aggregated proteins, altered cellular constituents, and molecules related to damaged neurons contribute to a cycle of inflammatory dysregulation. While genetic mutations, such as those affecting the amyloid precursor protein or presenilin in Alzheimer’s disease (AD) and parkin or UCHL1 in Parkinson’s disease, are known to trigger abnormal protein folding and aggregation, these mutations account for only ax subset of cases, indicating the involvement of additional factors in the accumulation of misfolded proteins such as amyloid-β (Wyss-Coray and Mucke, 2002). Among these factors, viral pathogens are increasingly recognized as potential contributors to neurodegenerative diseases. The ability of viruses to take control of cellular machinery and trigger inflammation is highlighted as one of the mechanisms driving degenerative processes. Additionally, they may directly induce protein misfolding and aggregation, hallmarks of these diseases (Leblanc and Vorberg, 2022). Oxidative stress caused by free radicals and lipid peroxidation during viral CNS infections is also highlighted as a key factor in the progression of neurodegeneration (Wyss-Coray and Mucke, 2002).

For instance, herpes simplex virus type 1 (HSV-1) is a highly prevalent virus that can remain latent in neurons, and its periodic reactivation is thought to act as a cofactor in AD (Letenneur et al., 2008). HSV-1 DNA is found in the brains of AD patients, particularly in those carrying the APOE4 gene. It has been identified in amyloid plaques, suggesting that reactivation of the virus may contribute to the formation of these plaques and the progression of AD (Wozniak et al., 2009). Similarly, certain viral outbreaks, such as influenza pandemics, have been linked to neurological conditions such as parkinsonism (Vilensky et al., 2010). Although many influenza virus strains do not directly infect the CNS, they trigger inflammatory responses, known as “cytokine storm,” which can lead to brain inflammation and neuronal damage. For instance, the 1918 Spanish flu pandemic was associated with post-encephalitic parkinsonism, and there are concerns that coronavirus disease 2019 (COVID-19), caused by SARS-CoV-2, could have similar long-term effects on the central nervous system (Xing et al., 2022).

In the case of multiple sclerosis (MS), recent studies have highlighted Epstein-Barr virus (EBV) as a potential cause. In a large cohort study, researchers found that individuals infected with EBV had a significantly higher risk of developing MS. In contrast, no such association was found with other viruses such as cytomegalovirus. Elevated levels of neurofilament light chain, a marker of neurodegeneration, were only observed after EBV infection, suggesting that this virus plays a crucial role in the development of diseases (Bjornevik et al., 2022). These examples illustrate a growing body of evidence that viral infections may contribute to the onset and progression of various neurodegenerative diseases, either by directly infecting the brain or by triggering harmful inflammatory responses.

Viral entry into the brain can occur via two main routes: the hematogenic route, through the blood–brain barrier (BBB), and the retrograde neuronal route. The BBB comprises molecular and cellular components, such as extracellular matrix, endothelial cells, pericytes, and astrocytes. Viruses can access the CNS through the BBB via three pathways: transcellular (infecting endothelial cells), Trojan horse (intracellular transport in infected immune cells), and paracellular (due to loss of BBB integrity) (Singh et al., 2021). Additionally, viruses can access the CNS via the retrograde neuronal route, through active axonal transport along peripheral neurons to the spinal cord, and by infecting olfactory neurons near the cribriform plate or choroid plexus epithelial cells (van Riel et al., 2015). When a virus invades host cells, it activates innate and adaptive immune responses. Specialized receptors present in host cells can recognize viral components through pattern recognition receptors. Among these receptors, Toll-like receptors (TLRs), retinoic acid-inducible gene I (RIG-I), nucleotide-binding oligomerization domain (NOD)-like receptors (NLRs), and C-type lectin receptors are notable. Activation of these pattern recognition receptors can result in the release of alarmins and inflammatory mediators aimed at promoting infection resolution (Thompson et al., 2011; Singh et al., 2021).

A key mediator in neuroinflammation is adenosine triphosphate (ATP), a crucial alarmine that activates inflammatory pathways during infection (Alves et al., 2020; Di Virgilio et al., 2020; Carvalho-Barbosa et al., 2023). ATP is maintained in millimolar concentrations in the intracellular environment but can be released into the extracellular space through compromised plasma membranes or various transporters, for example, membrane channels (such as pannexins or connexins), transport in vesicles or exocytosis from secretory granules (Dosch et al., 2018). Extracellular ATP and its enzymatic degradation products, such as adenosine diphosphate (ADP), adenosine monophosphate (AMP), and adenosine, can stimulate a variety of purinergic receptors present on the cell membrane (Giuliani et al., 2019). Purinergic receptors are classified into two main families: P1 and P2. The P1 family consists of G protein-coupled receptors activated by adenosine, which can be classified as either stimulatory (A_2A_ and A_2B_) or inhibitory (A_1_ and A_3_) receptors. The P2 family has two distinct subclasses, P2X and P2Y, which have affinities for nucleotides such as ATP, ADP, UTP, UDP, and UTP-glucose. P2Y family receptors (P2Y1, P2Y2, P2Y4, P2Y6, and P2Y11-14) are coupled to G proteins, while P2X receptors (P2X1-7) are ligand-gated ion channels that respond only to ATP (Fredholm et al., 2011; Jacobson et al., 2020; Aminin and Illes, 2021). Ectonucleotidases regulate the metabolism of extracellular nucleotides, converting ATP into ADP, AMP, and eventually adenosine, a molecule with immunosuppressive effects (Yegutkin, 2008). In the neuroimmune context, the main ectonucleotidases are E-NTPDases, such as CD39 (E-NTPDase1), NTPDase2 (CD39L1), and NTPDase3 (CD39L3), which catalyze the conversion of ATP and ADP into AMP, and 5’-nucleotidase (CD73), which converts AMP into adenosine (Alves et al., 2020). These enzymes are located on the cell surface or within the intracellular milieu and play important roles in modulating inflammation and neurodegenerative conditions (Álvarez-Sánchez et al., 2019; Meng et al., 2019).

Purinergic signaling plays a crucial role in regulating immune responses, acting through purinergic receptors on both peripheral immune cells, such as macrophages and lymphocytes, and the CNS, which influences microglial function. Receptors such as P2X4, P2X7, P2Y_4_, P2Y_6_, and P2Y_12_, as well as A_2A_ and A_3_ receptors, are located in microglia in the CNS (Illes et al., 2020). These receptors modulate various microglial processes, including motility, migratory activity, phagocytosis or pinocytosis, and the release of inflammatory mediators, such as pro-inflammatory cytokines, chemokines, growth factors, and reactive oxygen species (Illes et al., 2020; Morillas et al., 2021). Extracellular ATP activation of the P2X7 receptor on microglia causes a transient increase in Ca^2+^ and K^+^ efflux, resulting in the activation of the NLRP3 inflammasome and caspase-1, which promotes the production of pro-inflammatory cytokines such as interleukin (IL)-1β and IL-18. Extracellular ATP also activates P2Y_1_ and P2Y_6_ receptors in astrocytes, stimulating the production of arachidonic acid, prostaglandin E2, and nitric oxide, which are essential for inflammation. Additionally, high ATP concentrations activate P2X7 receptors, opening pores that increase the release of ATP and glutamate (Sidoryk-Węgrzynowicz and Strużyńska, 2021). Thus, P2 receptors play an important role in the pathology of neurodegenerative and infectious diseases affecting the CNS (Alves et al., 2020).

Therefore, we discuss the critical interplay between viral infections and neurodegenerative diseases, emphasizing how neuroinflammation is a pivotal mechanism in this relationship. The evidence presented underscores the role of various viruses in not only triggering inflammatory responses but also in potentially exacerbating neurodegenerative processes, often through mechanisms that disrupt immune regulation. Central to this dynamic is purinergic signaling, which acts as an important pathway linking viral infections to neurodegeneration by regulating immune responses and microglial function in the CNS. The activation of purinergic receptors by extracellular nucleotides modulates inflammation and influences the progression of neurodegeneration, highlighting the importance of these pathways for developing effective therapies to mitigate the impact of viral infections on the CNS.

## Search Strategy

PubMed was searched for relevant articles published from inception to 2024 using keywords such as “CNS viral infection,” “ATP viral infection,” purinergic signaling viral infection,” “purinergic signaling neurodegeneration,” “purinergic signaling CNS infection,” “neurodegeneration viral infections,” “neurodegeneration ATP,” and “neurodegenerative disease ATP.” The search results were manually screened based on the title and abstract. Articles on infections that did not happen on the central nervous system, and/or were not elicited by viral agents, and/or did not involve any purinergic signaling component, and/or did not induce neurodegeneration were excluded. More than 90% of the articles were published in the last 15 years (2010–2025).

## Human Immunodeficiency Virus

HIV is a multi-subtype retrovirus, expert in mutation, and the causative agent of the pandemic acquired immunodeficiency syndrome, with almost 40 million people living with the virus at the end of 2023 (Bbosa et al., 2019; World Health Organization, 2024a). HIV is transmitted through contact with infected blood, semen, vaginal fluids, and breast milk, most commonly via unprotected sex or sharing needles (Ghosn et al., 2018). HIV proteins such as Tat (Trans-Activator of Transcription) and gp120, essential for the virus replication and infection processes, are known to be closely involved with HIV-mediated neurocognitive disorders, as they can induce BBB dysfunction and disrupt dendritic and axonal integrity by undermining microtubules stability, leading to activation of multiple cell death pathways, among others (Fitting et al., 2015; Wenzel et al., 2019; Thompson et al., 2024). Thus, one of the many consequences of HIV infection is the development of HIV-associated neurocognitive disorder, which can ultimately progress to its worse form, HIV-associated dementia (Thompson et al., 2024).

**Figure 1:**
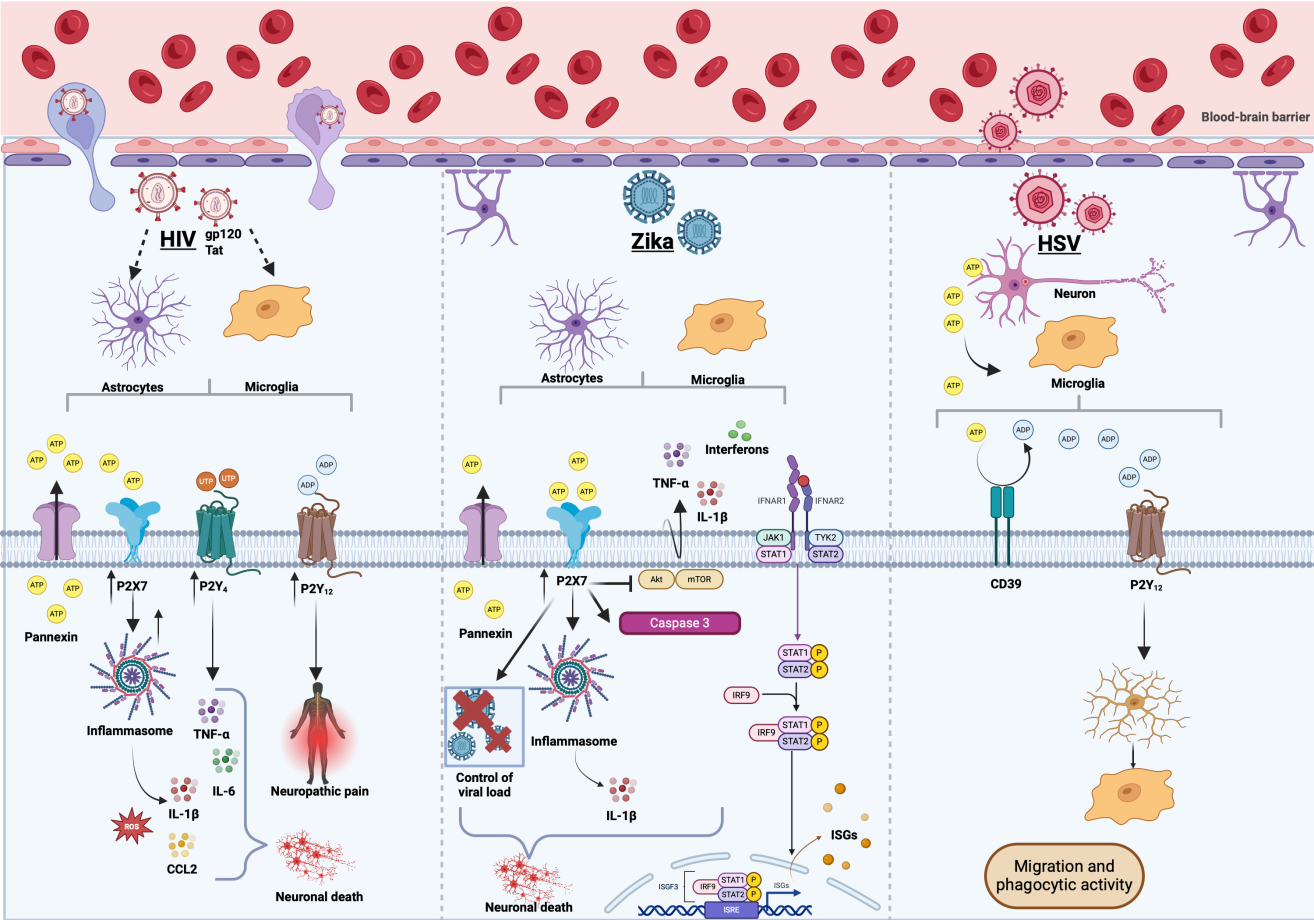
Schematic illustration showing mechanisms by which the purinergic signaling may trigger neuroinflammation and neurodegeneration in viral infections that affect the CNS, such as HIV, Zika, and herpesvirus. HIV infection modulates purinergic signaling receptors, stimulating the release of alarmins, such as ATP, through the pannexin hemichannel. The Tat and gp120 HIV proteins increase the expression of P2X7 and P2Y4 receptors in astrocytes, promoting the release of inflammatory molecules such as IL-1β, TNF-α, IL-6, CCL2, and reactive oxygen species, which contribute to neuronal death. Additionally, P2Y12 and P2X7 receptors are upregulated in glial cells and neurons, playing an important role in the development of HIV-induced neuropathic pain. Zika virus infection increases P2X7 receptor expression, which contributes to neuroinflammation, increasing the expression of glial markers GFAP (astrocytes) and IBA-1 (microglia). Although the P2X7 receptor is associated with neurodegeneration and neuroinflammation, mice expressing P2X7 showed more effective control of cerebral viral load than P2X7-deficient mice (P2X7^-/-^). This phenomenon may be explained by a more pronounced induction of inflammatory cytokines such as TNF-α, IFN-β, and ISGs such as IFIT-1, ISG15, and OAS2. However, the exacerbated release of these inflammatory molecules may contribute to neuronal death through caspase-3 activation and inhibition of the AKT/mTOR pathway. In the context of HSV-1 infection in neuronal cells, the release of nucleotides, such as ATP, activates microglia. The hydrolysis of ATP by purinergic enzymes, such as ectonucleotidase CD39, generates ADP, which activates P2Y12 receptors on microglia, inducing their migration and phagocytic activity. Created with BioRender.com. ADP: Adenosine diphosphate; AKT: protein kinase B; ATP: adenosine triphosphate; BBB: blood–brain barrier; CCL2: monocyte chemotactic protein-1; CD39: cluster of differentiation 39; CNS: central nervous system; gp120: envelope protein; GFAP: glial fibrillary acidic protein; HIV: human immunodeficiency virus; HSV-1: herpes simplex virus type 1; IBA-1: ionized calcium binding adaptor molecule 1; IFN: interferon; IFNAR: interferon alpha and beta receptor; IL-1: interleukin 1 beta; IL-18: interleukin-18; IL-6: interleukin-6; IRF9: interferon regulatory factor 9; ISGs: interferon-stimulated genes; ISRE: interferon-sensitive response element; JAK: Janus kinase; mTOR: mammalian target of rapamycin; NLRP3: NOD-like receptor protein 3; P: phosphorus; ROS: reactive oxygen species; STAT: signal transducer and activator of transcription protein; Tat: trans-activator of transcription; TNF-α: tumor necrosis factor-alpha; UDP: uridine diphosphate; UTP: uridine triphosphate.

In most studies to date, P2 receptors seem to be modulated by HIV infection to maintain its presence within the cell of the host (Carvalho-Barbosa et al., 2023). As extensively described, ATP may be released by endangered cells as an alarmin to neighboring cells. On that note, HIV-encoded envelope glycoprotein complex can stimulate the release of ATP by pannexin-1 channels from human peripheral blood mononuclear cells, stimulating purinergic transmembrane receptors, such as P2Y_2_, which contributes to viral replication (Séror et al., 2011). In human fetal astrocytes differentiated from neural precursor cells, for example, the single Tat protein was able to increase P2X7 receptor expression, leading to the release of pro-inflammatory molecules, such as monocyte chemoattractant protein-1 and CCL2 (Tewari et al., 2014). Although the protein promotes the survival of challenged astrocytes, it promotes cell death in a culture of primary human neurons (Tewari et al., 2014). Another P2 receptor influenced by Tat protein in a culture of primary mouse astrocytes is the P2Y_4_ receptor, whose expression was upregulated by this protein challenge, regulating inflammatory responses such as the release of tumor necrosis factor-alpha (TNF-α), IL-6, and monocyte chemoattractant protein-1, leading to apoptosis. In an *in vivo* model, P2Y_4_ knockout mice challenged with Tat showed decreased cell death and inflammation, reinforcing the role of this purinergic receptor Tat-induced neuroinflammation (Zhou et al., 2019; **Figure 1**).

Neuropathic pain in people with HIV is a common and debilitating condition that arises from nerve damage caused by the virus or as a side effect of certain older HIV antiretroviral therapies from drugs such as stavudine or didanosine. This pain is often chronic and can manifest as burning, tingling, or shooting sensations, typically in the hands and feet (a condition known as HIV-associated peripheral neuropathy) (Lu et al., 2021). As P2Y_12_ receptors expressed in satellite glial cells in the dorsal root ganglia are often associated with pain transmission, its involvement in gp120-induced neuropathic pain was evaluated in male Sprague-Dawley rats, showing that relative expression of P2Y_12_ receptor was increased in this model. Its downregulation with RNA interference technique (the short hairpin RNA, shRNA) relieved mechanical and thermal hyperalgesia following the gp120 challenge, forms of heightened sensitivity to pain commonly associated with neuropathic pain (Yi et al., 2018). In the same rat model, the P2X7 receptor was upregulated, and its antagonist, Brilliant Blue G (BBG), downregulated the receptor expression and the hyperalgesia (Wu et al., 2017).

The accumulation of amyloid-beta plaques and neurofibrillary tangles originating from intracellular aggregates of hyperphosphorylated tau protein are the hallmark features of AD (Zhang et al., 2021a). HIV-associated neurocognitive disorder may present cognitive impairments that can, in some cases, resemble AD, such as similar concentrations of beta-amyloid plaques and neurofibrillary tangles, especially as patients age (Canet et al., 2018; Chemparthy et al., 2021). The exact mechanisms linking these two conditions are not fully understood. Still, intriguingly enough, it was described that the HIV glycoprotein gp120, crucial for viral invasion and responsible for contributing to neuroinflammation, elevates P2X7 receptor expression in BV2 microglial cells (Chen et al., 2016; Liu et al., 2017b).

The overstimulated TLR4/MyD88/nuclear factor-kappa B signaling pathway in activated pro-inflammatory microglia can lead to chronic neuroinflammation and is implicated in the progression of various neurodegenerative diseases, including AD and HIV-associated neurocognitive disorder (Jones and Kounatidis, 2017; Freeman and Swartz, 2020; Singh and Singh, 2020; Zhou et al., 2020). Toll-like receptor 4 (TLR4) activated by pathogen-associated molecular patterns or damage-associated molecular patterns recruits the adaptor protein MyD88, which in turn triggers a signaling cascade leading to the activation of nuclear factor-kappa B. This transcription factor stimulates the translation of many pro-inflammatory cytokines, chemokines, and other intracellular molecules, such as inflammasomes (Liu et al., 2017a). This pathway is considered the first signal for the expression of NLRP3 inflammasome, and the second signal can be promoted by the P2X7 receptor activation. After P2X7 receptor activation by ATP, this complex is activated, mobilizing pro-caspase-1 and cleaving it into its active form, caspase-1, which in turn finalizes the activation of pro-IL-1β into IL-1β, driving inflammation (Di Virgilio et al., 2017). In AD, the blockade of the P2X7-ATP axis may be a potential target to prevent NLRP3 inflammasome final activation, IL-1β release, and consequent neuroinflammation (Thawkar and Kaur, 2019).

Similarly, in BV2 microglial cells challenged with gp120, the P2X7 receptor signaling cascade promoted the activation of nuclear factor-kappa B and the release of many inflammatory markers, such as TNF-α, IL-1β, and nitric oxide (Chen et al., 2016). This process contributed to the loss of microglial cells and resulted in neurological changes, including higher signals of HIV-1-associated dementia and the worst progression of HIV-associated neurocognitive disorder, such as memory loss and cognitive decline (Liu et al., 2017b). Of note, detailed methodologies and techniques for evaluating P2X7 and P2X4 receptors in microglial cells can be found in Leite-Aguiar et al. (2024a) and Sluyter et al. (2024), respectively.

## Theiler’s Murine Encephalomyelitis Virus

Theiler’s murine encephalomyelitis virus is a single-stranded RNA virus belonging to the Picornaviridae family. It serves as a valuable model for studying viral-induced demyelinating diseases, such as MS in humans, as it causes chronic CNS inflammation, leading to motor impairments, paralysis, and axonal damage, with effects resembling human autoimmune conditions. Depending on the strain and the genetic background of the host, TMEV can trigger either acute encephalitis or a chronic progressive disease that mirrors demyelination (Gerhauser et al., 2019; Pike et al., 2022).

Microglia are crucial in responding to viral infections, including during TMEV infection. During such infections, microglia become activated and shift from their surveillance mode to an aggressive state aimed at eliminating the invading pathogens, with changes in phenotype and transcription profiles to phagocyte disrupted or dying brain cells through P2Y_12_ receptors activation (Chen et al., 2019). In the acute phase of viral encephalitis, this activation involves the release of inflammatory cytokines and reactive oxygen species aiming at viral clearance to prevent lethal outcomes. However, prolonged overactivation may contribute to neuroinflammation and neuronal damage, as the balance between adequate viral clearance and excessive inflammation is delicate (Waltl and Kalinke, 2022).

Certain strains of TMEV, particularly the ones that cause persistent infection, are known to trigger acute seizures during the early stages of infection and can lead to chronic epilepsy. This occurs due to the virus infecting neurons and inducing inflammation in the brain, which disrupts normal neuronal function. The resulting neuroinflammation and viral persistence can alter the excitability of neural networks (Stewart et al., 2010). Regarding that, purinergic signaling can be involved in a series of brain diseases encompassing seizure episodes, such as neonatal seizures and chronic epilepsy disease (Méndez et al., 2020; Alves et al., 2024).

Following an acute acquired insult to the brain, such as an infection within the CNS, there is an increased calcium entry and cell death that can trigger the release of neurotransmitters and proconvulsive cytokines, cytokines that promote neuronal excitability and thus, propagation of seizures, as well as an augmentation of neuroinflammation. Purinergic signaling plays a crucial role in this process, as purine mediators such as ATP may be released, stimulating the P2X7 receptor and feeding the calcium influx, the cell death mechanisms, and even the release of neurotransmitters and proconvulsive cytokines, boosting epilepsy gravity (Méndez et al., 2020).

The P2X7 receptor shows a critical role in seizure severity, influencing it in a cell-dependent manner: in neurons, the P2X7-ATP axis seems to protect mice brains from worsened forms of epilepsy with anti-convulsive effects, whereas in microglia, it aggravates seizure episodes with increased hyperexcitability. Moreover, P2X7 receptor relative expression is seen in neurons more than any other P2X receptor, and it is particularly active during long-standing epilepsy in inhibitory neurons. In one of these neurons, the GABAeric interneurons, P2X7 receptor overexpression could reduce seizure severity and frequency in various epilepsy models, driving this purinergic receptor as a potential therapeutic target for epilepsy concerning analysis in these neuronal subtypes (Alves et al., 2024).

In a C57BL/6J mouse model of TMEV infection, in which hippocampal damage and acute seizures with the possibility of progression to chronic epilepsy are classic features, microglia cells are fundamental to control viral load by activating adaptive immunity by T cells. Their absence firmly changes C57BL/6J mice phenotype to a typically SJL/J mouse strain with TMEV infection phenotype, with higher viral burden and chronic demyelination, potentially progressing to a neurodegenerative disease state (Waltl et al., 2018). Additionally, in a functional imaging study on activated microglia following TMEV infection in a mouse model of temporal lobe epilepsy, it was shown that P2Y_12_-expressing microglia fail to maintain its surveillance status in the acute phase of the infection, proven by deficits in ATP-induced calcium events (Wallis et al., 2024).

## Herpes Simplex Virus Type 1

HSV-1 is a virus primarily known for causing oral herpes, which leads to cold sores or fever blisters around the mouth. HSV-1 is a member of the *Herpesviridae* family, and it is highly contagious, spreading through direct contact with infected saliva or lesions. Beyond oral infections, HSV-1 can also cause ocular infections, and in some cases, it may lead to more severe conditions like encephalitis. Once infected, the virus remains dormant in nerve cells, and it can reactivate periodically, leading to recurrent episodes, a period when HSV causes fluid-filled blisters to appear on or around the genitals, mouth, or rectum (Zhu and Viejo-Borbolla, 2021).

In HSV-1-infected neuronal cells, nucleotides like ATP are released, which trigger and activate microglia. The ultimate hydrolysis of ATP by purinergic enzymes, such as the ectonucleotidase CD39, generates ADP, which activates P2Y_12_ receptors on microglia. This enhances their migration and phagocytic activity to remove damaged neurons before their cell membranes break down. This mechanism helps mitigate abnormal neuronal activity that could otherwise lead to HSV-1 encephalitis (Fekete et al., 2018; **Figure 1**).

Aside from viral encephalitis, HSV-1 might play a role in the onset or progression of AD, as it can remain dormant in the brain for years (Marcocci et al., 2020). In the context of amyloid-β (Aβ) accumulation in AD, one of its many detrimental effects is the induction of ATP release into the extracellular space (Ronning et al., 2023). As neurons and glial cells are exposed to increasing levels of Aβ, they can become stressed or damaged, leading to the release of ATP as an alarmin that can hence activate purinergic receptors, particularly the P2X7 receptors, leading to the release of various pro-inflammatory cytokines, especially IL-1β, and the production of reactive oxygen species such as hydrogen peroxide by microglial cells (Parvathenani et al., 2003; Woods et al., 2012). This response contributes to chronic neuroinflammation, exacerbating neuronal damage and promoting the progression of Alzheimer’s pathology, and P2X7 receptor pharmacological inhibition by Brilliant Blue G (BBG) was defensive in restoring blood-brain barrier integrity and glial cells homeostasis (Ryu and McLarnon, 2008). Furthermore, as the P2X7 receptor is a type of ion channel that allows the influx of calcium ions (Ca^2+^) into the cell, its overactivation by the excessive ATP release can raise cytoplasmic Ca^2+^ levels, resulting in mitochondrial Ca^2+^ overload, which impairs organelle function and can lead to neuronal stress with triggered reactive oxygen species production and pro-apoptotic factors release, ultimately contributing to cell death (Berridge, 2011; Merighi et al., 2021).

The two enzymes responsible for the neuroinflammatory Aβ plaque formation, β-secretase, and γ-secretase have their cleavage activity of the amyloid precursor protein (APP) into Aβ stimulated by the kinase glycogen synthase kinase 3. Their activity is opposed to the α-secretase enzyme, which cleaves APP within the Aβ region, producing a soluble fragment called sAPPα, which is neuroprotective and supports synaptic function (Cieślak and Wojtczak, 2018). P2X7 receptor activation decreases α-secretase activity by promoting glycogen synthase kinase 3 activity, and the inhibition of either native or overexpressed P2X7 receptor resulted in consequent inhibition of glycogen synthase kinase 3 activity, leaving the way clear for the α-secretase neuroprotective role (Diaz-Hernandez et al., 2012). Another mechanism by which ATP signaling may contribute to the progression of AD is the production of H_2_O_2_ triggered by activating the P2X7 receptor. In activated rat microglia, the signaling cascade downstream from this receptor leads to the activation of the NADPH oxidase enzyme and subsequent activation of the p38 MAP kinase pathway, releasing superoxide and establishing a neurotoxic setting positively correlated to plaque aggregation in a transgenic model of AD (Parvathenani et al., 2003).

## Epstein-Barr Virus

The EBV is a member of the herpesvirus family and one of the most common human viruses worldwide. It primarily spreads through saliva and is the cause of infectious mononucleosis, often referred to as “mono” or the “kissing disease.” EBV is also associated with several other illnesses, including certain cancers such as Burkitt’s lymphoma, nasopharyngeal carcinoma, and Hodgkin lymphoma, as well as autoimmune diseases such as MS – EBV antibodies are often found at elevated levels in MS patients (Bjornevik et al., 2023). Most people are infected with EBV at some point in their lives, often in childhood, when it typically causes mild or no symptoms. However, when contracted later in adolescence or adulthood, it can lead to more pronounced symptoms, including fever, sore throat, swollen lymph nodes, and fatigue. Once infected, the virus remains dormant in the body, potentially reactivating later in life, especially in individuals with weakened immune systems (Houen and Trier, 2021; Damania et al., 2022).

MS is a chronic autoimmune disease in which the immune system attacks the protective myelin sheath covering nerve fibers in the CNS. This damage disrupts nerve signals, leading to a range of symptoms such as muscle weakness, difficulty with coordination and balance, vision problems, and cognitive issues. MS can vary in severity and progression, with periods of relapse and remission (Dobson and Giovannoni, 2019).

Concerning EBV infection with MS development, ATP release in higher quantities may be pivotal in a model of two steps explaining neuroinflammation, the so-called two-hit hypothesis. It suggests that neurodegenerative diseases develop due to a combination of two factors or “hits.” The first hit is the injury itself, which could be caused by a genetic predisposition such as an autoimmune reaction, a pathogenic infection, a hypoxia-ischemia, and others, that leads to microgliosis and astrogliosis, which may lead to the second hit, which involves an abnormal neuronal release of ATP to the extracellular milieu following direct injury or indirect microglial/astroglial activation. Together, these hits lead to sustained neuroinflammation, contributing to the progression of neurodegenerative diseases (Fiebich et al., 2014; Levine et al., 2023).

In MS, microglia play a pivotal role in the pathology of diseases, as they become activated in response to myelin damage and inflammation, contributing to the progression of MS by further damaging myelin and nerve fibers. While microglia initially attempt to clear debris and repair damage, their prolonged activation can lead to excessive inflammation and neurodegeneration, exacerbating the symptoms of MS (Voet et al., 2019). The TRPV1 channel (transient receptor potential vanilloid 1), a receptor primarily found on sensory neurons that is known to be involved in pain perception and thermal sensation, is a calcium (Ca^2+^)-permeable channel that can also be expressed and highly activated in microglia cells of the anterior cingulate cortex, as well as mildly expressed/activated in the thalamus, somatosensory cortex, periaqueductal gray and hippocampus, working in detecting brain inflammation (Marrone et al., 2017). In C57BL/6 mice subjected to an experimental autoimmune encephalomyelitis model, one of the most common animal models to study MS, TRPV1 channels lead to an increase in calcium influx, augmenting ATP secretion, and then activating NLRP3 inflammasome and aggravating clinical outcomes of experimental autoimmune encephalomyelitis, which suggests that the ATP signaling pathway may be engaged in MS – microglia – EBV route (Zhang et al., 2021b).

## Dengue Virus

Dengue virus (DENV) is a mosquito-borne virus responsible for causing dengue fever, a potentially severe illness affecting millions worldwide, particularly in tropical and subtropical regions. Transmitted primarily by Aedes mosquitoes, mainly *Aedes aegypti*, DENV exists in four distinct serotypes (DENV-1, DENV-2, DENV-3, and DENV-4). Infection with one serotype provides lifelong immunity to that specific serotype but not to the others, which can lead to severe forms of the disease, such as dengue hemorrhagic fever or dengue shock syndrome, upon subsequent infections (Roy and Bhattacharjee, 2021; Kok et al., 2023). By the end of April 2024, over 7.6 million dengue cases have been reported to the World Health Organization in the four months of 2024, with more than 3000 fatalities. The majority of those cases happened in the Americas continent (World Health Organization, 2024b).

In DENV infection, a culture of primary human monocytes pre-treated with ATP had a lower viral load when infected with DENV, especially in monocytes positive for P2X7 receptor, evaluated by the antigen of the non-structural 1 DENV protein (NS1) (Corrêa et al., 2016). The ATP-P2X7 triggers the nitric oxide production in these cells, exercising its antiviral effects by reducing the levels of inflammatory chemokines and cytokines such as CCL2, CXCL10, IL-8, and TNF-ɑ in infected cells (Corrêa et al., 2016).

As direct viral invasion of the CNS or an exaggerated immune response in severe dengue cases may generate dengue-associated encephalitis, with symptoms as seizures, altered mental status, headaches, and even coma, there can be a role for the purinergic microglial receptor P2Y_12_, as it has been described that microglia protect from viral replication as well, since its pharmacological depletion by clodronate liposomes in DENV infection reduced antiviral cytokines production, leading to increased brain viral titer, worsen clinical scores and elevated mortality (Tsai et al., 2016). Nevertheless, studies have not established a relationship between DENV infection, purinergic signaling, and neuroinflammation.

## Zika Virus

Zika virus (ZIKV) is a mosquito-borne flavivirus primarily transmitted by *Aedes* mosquitoes, notably *Aedes aegypti* and *Aedes albopictus*. First identified in Uganda in 1947, ZIKV gained global attention during the 2015–2016 outbreak in the Americas. It is typically spread through mosquito bites but can also be sexually or transvertically transmitted (Giraldo et al., 2023). Despite its relatively mild symptomatology in most cases, the association with severe congenital disabilities and neurological issues has made ZIKV a significant public health concern, as it has been deeply associated with severe congenital disabilities such as microcephaly in newborns, being a manifestation of congenital Zika syndrome (Costello et al., 2016). Neurological complications associated with Zika virus infection are not limited to congenital cases, as adult infection can lead to a variety of conditions, including encephalitis, encephalomyelitis, acute transverse myelitis, as well as the autoimmune Guillain-Barré syndrome (Cao-Lormeau et al., 2016; Muñoz et al., 2016).

The immediate and lasting effects of Zika virus infection were further studied in postnatal day 3 Swiss mice to investigate the long-term impacts of ZIKV infection during the perinatal period, which exhibited a strong preference for brain tissue, resulting in microcephaly development after birth, along with several behavioral impairments in adulthood. During the initial infection phase, the mice frequently experienced seizures, which were lessened through inhibition of TNF-α. In adulthood, ZIKV continued to replicate in mice infected as neonates, leading to greater vulnerability to chemically induced seizures, neurodegeneration, and brain calcifications (De Oliveira Souza et al., 2018). Indeed, TNF-α signaling was closely involved in memory loss and brain inflammation in human and mouse adult brain tissue infected with ZIKV (Figueiredo et al., 2019), and TNF-α blockade by its monoclonal antibody Infliximab was enough to prevent ZIKV-induced cognitive and motor impairment in Swiss mice 60 days post-Zika infection, with reduced brain cell death (Christoff et al., 2024).

Nevertheless, it is important to be vigilant about certain immunosuppressive treatments, as ZIKV-infected mice that received immunosuppressant drugs, such as dexamethasone, have already shown an increased susceptibility to chemically induced seizures. They also displayed increased levels of subgenomic flavivirus RNAs (sfRNAs) associated with altered cytokine expression, such as decreased interferon (IFN)-β and increased IL-1β expressions (Nogueira et al., 2024).

In young adult mice deficient in TRIF and IPS-1, the adaptor proteins of TLR3 and RIG-I/MDA-5, respectively (components of the immune system that recognize viral RNA and lead to the production of interferons and other antiviral responses), ZIKV infection also increased the levels of many cytokines 7 days post-infection, such as IL-1α, IL-6, IL-9, IL-10, IL-12p70, and IFN-γ (Enlow et al., 2021). Zika antigens were found in various regions within the brain, especially in microglial cells in the dorsal hippocampus with high phagocytic activity, and drug-induced deficiency by brain-penetrant CSFR1 inhibitor PLX5622 in ZIKV infection context also increased brain viral load and ZIKV^+^ percentage of neurons and astrocytes (Enlow et al., 2021). Moreover, its depletion showed increased phagocytic activity from astrocytes, indicating that this cell type may partially take over the task of removing ZIKV-infected cells through phagocytosis (Enlow et al., 2021). Neonatal ZIKV infection in mice led to postnatal microcephaly, persistent behavioral issues, and increased vulnerability to neurodegeneration and seizures in adulthood. Findings suggest that early TNF-α inhibition could help prevent chronic neurological effects.

Even though no connection has been made between adult ZIKV-associated neurological complications and purinergic signaling, recently, the role of the pro-inflammatory ATP-P2X7 axis concerning Zika virus-associated brain anomalies has been investigated in newborn mice. The increased expression of the P2X7 receptor in the brains of ZIKV-infected newborn mice is prominent compared to the non-infected mock group. Hippocampal analyses indicated that the P2X7 receptor expression exacerbated damage in the CA1/CA2 and CA3 infected subregions. Furthermore, a significant reduction in the neuronal migration protein doublecortin expression was observed in the brains of ZIKV-infected mice. As seen with the model of genetic depletion of the P2X7 receptor, wild-type mice showed worsened motor performance in infected mice, as well as increased expression of glial markers glial fibrillary acidic protein (GFAP) (astrocytes) and ionized calcium binding adaptor molecule 1 (IBA-1) (microglia). Although the P2X7 receptor is involved in neurodegeneration and neuroinflammation, wild-type mice demonstrated better control of brain viral load compared to P2X7^-/-^ mice, possibly due to more significant induction of TNF-α, IFN-β, and interferon-stimulated genes (ISGs), such as IFIT-1, ISG15, and OAS2. The P2X7 receptor inhibits the neuroprotective protein kinase B/mammalian target of rapamycin (AKT/mTOR) pathway, and it also promotes caspase-3 activation, suggesting that it may have distinct roles in neurodegeneration and that modulatory therapies targeting P2X7 may be useful in reducing neurological complications of ZIKV (**Figure 1**; Leite-Aguiar et al., 2024b).

Nongenomic long-chain RNAs, called subgenomic or non-coding RNAs, are produced alongside the genomic RNA during flavivirus infection. These include subgenomic flavivirus RNAs (sfRNAs), a specific type of viral long non-coding RNAs (lncRNAs). SfRNAs are particularly significant because they play critical roles in viral persistence and pathogenesis, acting as the primary lncRNAs involved in modulating host-pathogen interactions and sustaining the infection (Pijlman et al., 2008; Slonchak and Khromykh, 2018; Slonchak et al., 2022; Mattick et al., 2023). These sfRNAs, resistant to degradation, interfere with host immune responses by modulating inflammatory pathways, such as the IFN-β signaling, and contribute to the apoptosis of neural progenitor cells. Evidence highlights the pivotal roles of human lncRNAs in regulating neuronal function, synapse stability, and synaptic plasticity (Samaddar and Banerjee, 2021; Wang et al., 2021). Recently, it was demonstrated that neonates infected with ZIKA exhibit an enhanced generation of sfRNAs in the brain when subjected to immunosuppression in adulthood. This increase correlates with altered expression of inflammatory mediators and heightened susceptibility to seizures, suggesting that viral lncRNAs play a role in sustaining neuroinflammatory and neurodegenerative processes well beyond the acute phase of infection (Nogueira et al., 2024). Additionally, these lncRNAs can modulate inflammatory and immune responses by influencing purinergic signaling pathways, which include cytokine secretion and reactive oxygen species formation (Wu et al., 2019). These combined effects, including persistent epigenetic changes induced by lncRNA, could have long-term implications for neurological health, even after the virus is cleared (Mattick et al., 2023). Understanding the role of ZIKV non-genomic RNAs in these processes is crucial for elucidating the mechanisms underlying virus-associated neurodegeneration and developing effective therapeutic strategies.

## Vesicular Stomatitis Virus

Vesicular stomatitis virus (VSV) is a virus from the *Rhabdoviridae* family, closely related to the rabies virus, and is known to primarily affect livestock, including cattle, horses, and pigs. It causes vesicular disease, which is characterized by the formation of blisters and ulcers in the mouth, nostrils, and hooves, similar to symptoms of foot-and-mouth disease. While primarily an animal pathogen, VSV can occasionally infect humans, leading to a mild, flu-like illness. The virus is typically transmitted through direct contact with infected animals or via insect vectors such as sandflies and blackflies (Pelzel-McCluskey, 2023).

In a VSV model, active microglia were shown to accumulate in the olfactory bulb, one of the main getaways for the CNS. Their accumulation is a result of the strong signaling of IFN-1 through type I IFN receptors in neurons and astrocytes, which stimulates microglia protective state in creating a barrier against viral invasion (Chhatbar et al., 2018). Indeed, the IFN-1 signaling seems to be very important in the microglia role in viral encephalitis, as the downstream genes stimulated by it – ISGs – may be produced by microglia themselves or by surrounding cells (Chen et al., 2019). As mentioned above, the phagocytic phenotype of microglia may be heavily induced by P2Y_12_-ATP axis. This seemly barrier that microglia create around infected neurons may be closely dictated by P2Y_12_ receptor-dependent mechanisms, as ATP in micromolar concentrations can be released by pseudorabies virus-compromised neurons, then degraded to ADP, which activates microglial P2Y_12_ receptors that stimulate the migration of these cells to the area of damage to try to intercept transneuronal viral transmission (Fekete et al., 2018).

## Severe Acute Respiratory Syndrome Coronavirus 2

SARS-CoV-2 is the virus responsible for COVID-19, a global pandemic that began in late 2019. It is a coronavirus that primarily spreads through respiratory droplets from coughs, sneezes, or talking. SARS-CoV-2 infects human cells by binding to the ACE2 receptor, leading to symptoms that range from mild respiratory issues to severe illness, including pneumonia and acute respiratory distress syndrome. The virus can also affect other systems, leading to complications such as cardiovascular, renal, and neurological problems (Hu et al., 2021; Gusev et al., 2022).

**Figure 2:**
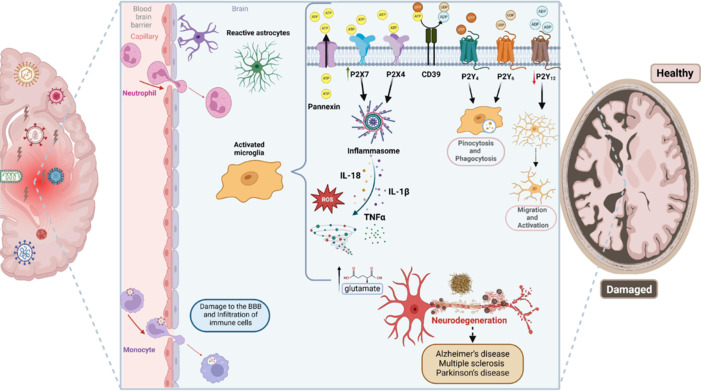
Schematic illustration showing purinergic signaling mechanisms contributing to neuroinflammation and neurodegeneration induced by neurotropic viruses. Viral entry into the brain can occur via two main routes: hematogenous (through the BBB) and retrograde neuronal. In the hematogenous route, viruses can access the CNS through three mechanisms: transcellular, by infecting the endothelial cells of the BBB; “Trojan horse,” by intracellular transport in infected immune cells; and paracellular, due to the loss of BBB integrity. Viral invasion triggers the release of alarmins, such as ATP, which activates purinergic signaling in glial cells, particularly in microglia. ATP is released via exocytosis and channels such as connexins and pannexins. Subsequently, ATP can be hydrolyzed to ADP by ectonucleotidases, such as CD39. Activation of purinergic receptors, such as P2X7, can induce NLRP3 inflammasome activation and release pro-inflammatory cytokines (IL-1β and IL-18), chemokines, growth factors, ROS, and glutamate. Accumulation of glutamate can induce excitotoxicity and neuronal death. P2Y_12_ receptor activation promotes the extension of microglial processes in response to CNS damage, while negative regulation of P2Y_12_ and positive regulation of P2X4 control the retraction of these processes, guiding microglial migration. Finally, the amoeboid phenotype of microglia induces phagocytosis and pinocytosis through the activation of P2Y_6_ and P2Y_4_ receptors, respectively. This chronic inflammatory environment, sustained by a dysregulated immune response and the excessive release of toxic molecules, may serve as the link between viral infection in the CNS and the development of neurodegenerative diseases. The persistent activation of microglia and the continuous release of inflammatory mediators can promote neuronal death, synapse loss, and brain dysfunction, which are key features of several neurodegenerative conditions such as Alzheimer’s disease, Parkinson’s disease, and ALS. Thus, purinergic signaling and the inflammatory response associated with viral infection are not only crucial for immune defense but also play a central role in the pathogenesis of neurodegenerative diseases. Created with BioRender.com. ADP: Adenosine diphosphate; ALS: amyotrophic lateral sclerosis; AMP: adenosine monophosphate; ATP: adenosine triphosphate; BBB: blood–brain barrier; CD39: cluster of differentiation 39; CNS: central nervous system; Glu: glutamate; IL-1: interleukin 1 beta; IL-18: interleukin-18; NLRP3: NOD-like receptor protein 3; ROS: reactive oxygen species; TNF-α: tumor necrosis factor-alpha; UDP: uridine diphosphate; UTP: uridine triphosphate.

Considering the recent devastating pandemic of COVID-19, there have been plenty of studies showing that the causative virus, the virus SARS-CoV-2, can cause neuroinflammation and damage to the brain, with many neurological outcomes, such as cognitive decline, memory issues, and others which may accelerate neurodegenerative processes (Spudich and Nath, 2022). The immune response of the host may have a severe immune reaction by releasing excessive cytokines to halt infection, known as a “cytokine storm.” This excessive immune response leads to widespread inflammation and tissue damage. It can result in the activation of the pro-inflammatory P2X7-ATP axis, intensifying this inflammatory milieu and exacerbating or triggering Alzheimer’s-related pathology (**Figure 2**), particularly in those who are genetically predisposed (Ribeiro et al., 2021). Interestingly, increased ATP levels in the serum of COVID-19 patients were detected (Vicentino et al., 2023).

The SARS-CoV-2 spike protein is a viral protein that enables the virus to bind to and enter human cells by interacting with the ACE2 receptor. When infused directly into the lateral ventricle, this protein can solely cause synapse damage, memory impairment, and neuroinflammation in adult mice (between 30 and 45 days after spike injection), as well as the total SARS-CoV-2 virus (Fontes-Dantas et al., 2023). However, this effect is diminished once TLR4 signaling is genetically or pharmacologically depleted, suggesting the involvement of Toll-like receptor 4 signaling in COVID-19 cognitive dysfunction (Fontes-Dantas et al., 2023).

The Spike protein is capable of inducing ATP secretion in a microglia cell line, upregulating a range of purinergic components, both receptors and ectonucleotidases, such as P2Y_6_, P2Y_12_, NTPDase2, and NTPDase3. Immunofluorescence analysis also showed that P2X7, P2Y_1_, P2Y_6_, and P2Y_12_ protein levels were higher in BV2 cells after spike protein infusion. (Alves et al., 2023). In the same study, 8 to 12 weeks Swiss mice intracerebroventricular Spike infusion was performed as previously described (Fontes-Dantas et al., 2023), and their hippocampus also showed high relative levels of P2X7, P2Y_1_, P2Y_6_, P2Y_12_, NTPDase1, and NTPDase2, and immune analysis further confirmed high expression of the P2X7 receptor in microglial cells in some specific hippocampal areas, such as the CA3/DG regions (Alves et al., 2023). There has been a myriad of research showing the relevance of microglia cells in COVID-19 disease *in vivo* as well, such as an analysis of deceased German patients infected with SARS-CoV-2 that displayed activation of microglia cells, especially in the brainstem and cerebellum of more than 80% of patients analyzed post-mortem (Matschke et al., 2020).

## Conclusions

ATP functions as a pivotal alarmin in the context of neurotropic viral infections, playing a crucial role in mediating neuroinflammation and neurodegeneration. Upon infection, neurotropic viruses can trigger the release of ATP from damaged or stressed neural cells, which acts as a danger signal that activates various immune responses. This extracellular ATP interacts with purinergic receptors on microglia and other immune cells, initiating a cascade of pro-inflammatory signals (Carvalho-Barbosa et al., 2023). While this response is essential for controlling the viral infection, excessive or prolonged inflammation can lead to detrimental effects on neural tissue, contributing to neurodegenerative processes (**Figure 2**).

The dual role of ATP in neuroinflammation underscores the delicate balance required between protective immunity and neurotoxicity. ATP-driven immune activation aids in the clearance of viral pathogens, while on the other hand, its persistent signaling can exacerbate neural damage through sustained inflammatory responses (Savio et al., 2018). Understanding how ATP and purinergic signaling pathways contribute to these processes offers valuable insights into the mechanisms underlying neurodegeneration observed in viral infections. Additionally, it highlights potential therapeutic targets, such as modulating purinergic receptors, to mitigate harmful inflammation without compromising the essential antiviral response.

This review highlights the extracellular nucleotides as critical alarmins in neurotropic viral infections and emphasizes the complex interplay between the nervous and immune systems. Targeting ATP-mediated signaling pathways could represent a promising strategy for developing novel treatments to reduce neuroinflammation and prevent long-term neurodegenerative outcomes. As research continues to unravel these mechanisms, there is hope for new therapeutic interventions that can effectively address the neuroinflammatory and neurodegenerative consequences of neurotropic viral infections.

## Data Availability

*Not applicable*.
